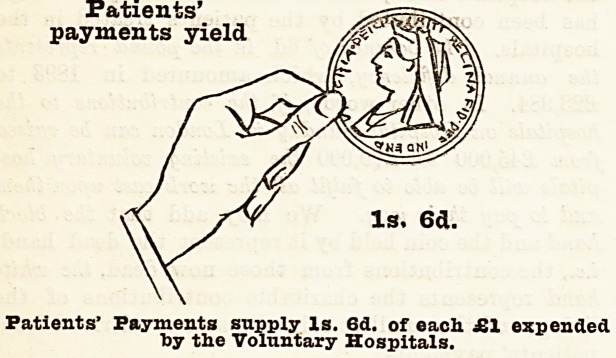# Special Hospital Sunday Supplement

**Published:** 1895-06-15

**Authors:** 


### Special Hospital Sunday Supplement.

(Figure 1.)

The Population of Londo :, 4,306,411.

### The Proportion of Diseases to Population in London.

With a view of endeavouring to bring home to the minds of everybody what illness means to the people of London, we have prepared two diagrams representing Father Thames standing in a boat with St. Paul's and the City of London behind him. These diagrams are drawn to scale, and represent exactly the proportion which disease (Figure 2) represents to population (Figure 1). They are based upon a calculation of the whole population compared with the actual number of people treated in the London voluntary hospitals, including St. Bartholomew's, Guy's, and St. Thomas's, and dispensaries, and the Metropolitan Asylums Board hospitals in 1893. From a return which we have received from the Registrar-General it appears that the population in the administrative county of London in 1893 was 4,306,411, of which 1,136,884 were men over twenty, 1,340,288 were women over twenty, and 1,829,239 were children, *i.e.*, boys and girls under twenty years of age. These 4,306,411 persons are represented by the figure of Father Thames in the left hand column at the top. In this connection it may be interesting to state that of the whole population affected, 38·2 per cent. who attended the hospitals were treated for medical diseases, 41·6 per cent. for surgical diseases, 1·3 per cent. were fever cases, 5·4 per cent. were eye cases, 2·5 per cent. suffered from diseases of the skin, 2·7 per cent. were cases of consumption, 2·8 per cent. suffered from some affection of the throat and ear, 5·1 per cent. were women admitted to the women's hospitals, and ·4 per cent. were cases of paralysis. The actual figures, of which the percentages are here given, will be found against the diagrams published on the two following pages. We may add that, in order to make the figures as far as possible comparable and accurate, where the cases treated at the general hospitals are analysed in the returns, the number of each class has been included under its proper head. The total number of the patients treated at the remaining hospitals has been distributed under the several heads, according to percentages based upon the analyses available.

### The Illnesses of Londoners.

Out of a population of upwards of four and a quarter millions, a very large proportion when ill must be able to pay for the services of a private medical attendant. Still no less than 1,717,187 people received treatment at the London voluntary hospitals and dispensaries, and those connected with the Metropolitan Asylums Board. It is fair to presume that the illnesses which attacked nearly one-half of the population which were treated at the hospitals were for the greater part something more serious than minor ailments. The precise proportion which illness represents to healthy Londoners each year will be seen by comparing the Figure 2 at the bottom of the right hand column on this page with Figure 1 at the top. We imagine that few people can have realised how serious are the ravages of illness in London in the present day. Disease is everywhere. It attacks the pauper and the peer; old and young; children and adults; none escape its ravages. It is, undoubtedly, an appalling fact that so large a proportion of the population should receive hospital relief. The picture would be even more serious were we able to add to it all the patients who when ill paid for their own treatment at the hands of one of the thousands of medical practitioners engaged in private practice within the administrative county of London. Let anyone try to picture London without hospitals. Let them realise for a moment the enormous additional increase in the number and severity of the illnesses which would then affect all classes of the community, and the £70,000 needed as a free gift on Hospital Sunday to enable the managers to maintain our voluntary hospitals with the utmost efficiency will surely be forthcoming, because everybody must then recognise the responsibility, not of giving to the hospitals, but of refusing to invest a small but necessary sum as a protection to themselves and their families against the added dangers of city life which would result to themselves from the absence of adequate hospital accommodation.

(Figure 2.)

A Year's Hospital Patients. Total, 1,717,187.

### DISEASES FROM WHICH LONDONERS SUFFER.

Diagrams of Diseases.

WITH a view of showing at a glance the nature of the illnesses from which the inhabitants of London suffer each year we have prepared the following diagrams. *Altogether one million seven hundred and seventeen thousand one hundred and eighty-seven* members of the population of London were treated for some form of illness at the London voluntary hospitals—including St. Bartholomew's, Guy's, and St. Thomas's—the metropolitan dispensaries, and the Metropolitan Asylums Board hospitals during the year 1893. The approximate number of patients treated for the various classes of diseases will be readily appreciated by a glance at the diagrams of diseases in the right hand column.

**Patients Suffering from Surgical Diseases.**—Of the whole number of patients received by the hospitals *six hundred and sixty-seven thousand and twenty-eight* required surgical treatment. What is meant by "surgical diseases"? They include all accidents, *i.e.*, broken bones, smashed limbs, fractured skulls, and all manner of fractures, displacements, and crushings of sensitive parts or organs. They further include abscesses, ulcerations, cancers, and tumours of all kinds; and, indeed, every injury which accident or pathological process may produce. Surgical diseases include all accidents and all lesions which may be dealt with by hand or instrument. Let anyone who desires to realise what surgical diseases mean for the population of London try to realise this army of 667,028 persons suffering from one or more of the injuries here briefly summarised.

**Patients Suffering from Medical Diseases.**—*Five hundred and eighty-four thousand seven hundred and sixty* cases received medical treatment. What does the medical profession mean by medical diseases? Diseases which are situated in their entirety or as to their source and origin in one or other of the three great cavities of the body, many of them deep-seated, most of them removed from sight, the diagnosis of their nature and extent is dependent upon the scientific knowledge of the doctor to whose treatment they are committed. They include typhoid, rheumatic, scarlet and other fevers, small-pox, measles, chicken-pox, pneumonia, pleurisy, bronchitis, every kind of heart disease, many forms of brain lesion, diseases of the stomach, bowels, liver, kidneys, bladder, pancreas and spleen, most nervous diseases, dyspepsia, constipation, headaches, sleeplessness, and a myriad other ailments, many of them serious, and often resulting in grave danger to life, or at least to the useful existence of mankind. Imagine for a moment considerably more than half a million human beings in London alone attended, free of cost to the patients, by the leading physicians of the day within the buildings of the hospitals of London.

**Patients Treated at Special Hospitals for Children.**—*One hundred and twenty-seven thousand four hundred and fourteen* young people grievously ill sent from homes where they could not be properly treated or even carefully attended to. The fine illustration which is presented with this special number of THE HOSPITAL will bring to the mind of every sympathetic person who studies it what children have to suffer, and how essential it is to the well-being of the race that they should have the skilled medical and surgical care which the hospitals can alone supply to the most grievous of the many cases which arise amongst a large population like that of the metropolis of the British Empire. Surely no words are needed to make the younger residents in this great city determine to give something, even from a limited income, to help suffering children to secure a return to health, and the power of one day shielding themselves against the dangers and risks to life of a vast city like London.

**Patients Suffering from Eye Affections.**—*Ninety-one thousand three hundred and twenty-four* persons are annually treated by the ophthalmic hospitals of London. It will be seen that, apart from the blind, there were nearly ten thousand people suffering from various forms of disease of the eye which often entailed excruciating pain, and must in many cases have terminated in loss of sight had it not been for the treatment they received at the hospitals. Those who are blessed with sight will surely give something to the hospitals on Hospital Sunday as a thank-offering for their escape from one of the most cruel diseases to which the human frame is liable.

**Diseases of Women and Motherhood.**—*Eighty-six thousand six hundred and twenty-two* women were treated at the hospitals for women and the lying-in institutions of the metropolis during the year. Sons of good mothers, and, indeed, all sons must necessarily be moved to pity by realising that, apart altogether from the diseases to which all are liable, woman has to face others which are peculiar to her sex and which entail an immensity of suffering, if they do not

### DISEASES FROM WHICH LONDONERS SUFFER—*continued.*

render the sufferer permanently disabled from the enjoyment of life and the pursuit of occupations which render life happy and profitable. This group of diseases must excite the sympathies of the most careless, and we make bold to believe that it will stir up many persons to give something this year to the hospitals if they have never yet enjoyed the luxury of investing a handsome sum in so noble a cause.

**Patients Suffering from Consumption.**—*Forty-six thousand seven hundred and ninety-nine* persons suffering from phthisis or consumption were treated at the consumption hospitals of London during the year. Consumption is the curse of our climate. It spares neither the peer nor the pauper. It attacks the young, the old, and the middle-aged, whilst its ravages defy the highest skill of the greatest physicians of modern times. Who can say with certainty that he may not fall a victim to this dire disease, and who can find it in his heart to deny something towards the cost of alleviating the sufferings of those who have been stricken with this terrible malady?

**Patients Suffering from Diseases of the Ear and Throat.**—*Forty-four thousand three hundred and twelve* persons were treated at the special hospitals devoted to these diseases. The ear, throat, and nose are intimately connected, and large numbers of people require a visit to one of the special hospitals devoted to the ailments included in this section. Residents in a city like London, including both children and adults, are specially liable to these affections, which may involve temporary or permanent impairment of hearing, swallowing, breathing, and even speaking. Consider what it would mean to any one of us to suffer from one or all of these affections. A little quiet reflection will surely awaken sympathy for those who thus suffer.

**Patients Suffering from Diseases of the Skin.**—*Thirty-nine thousand eight hundred and five* patients were treated at the special skin hospitals of London during the year. When it is remembered that many people of both sexes are terrified by the appearance of any affection of the skin, even in these days of advanced science, when immediate alleviation is possible, and permanent relief in the majority of cases assured, little need be said to awaken the sympathy of the reader for the many thousands who annually suffer from ailments of this description. We were asked this week by an anxious husband, who was away when his wife died of malignant small-pox, whether his beautiful wife was disfigured by the disease? This question is eloquent of the popular view of skin maladies, and we confidently claim a liberal offering from every inhabitant of London towards the funds needed to rescue those who have to suffer from the extremely common but essentially disagreeable illnesses included in this section.

**Patients Suffering from Fever.**—Few members of the adult population can have failed to have experienced an attack of fever of some kind in the course of their lives. In these days, thanks to vaccination, the majority escape small-pox which used to ravage the population, and which very often left its mark upon the features of the people. Had the *twenty-two thousand* people who suffered from this infectious disease during the year been allowed to remain scattered throughout the entire population of London, fever cases would have been increased from thousands to hundreds of thousands, with a proportionate death-rate which it is horrible to contemplate. Give, therefore, on Hospital Sunday as a mark of intelligent recognition of the powers of preventive medicine in the present day.

**Patients Suffering from Paralysis.**—*Seven thousand three hundred and nineteen* people were stricken with paralysis and received treatment at the several hospitals devoted to this malady. In the morning a man rises from his couch in robust health, and leaves his home to pursue his ordinary avocations. Whilst he is in the counting-house, or the study, or the office, or in the shop following his ordinary business, paralysis seizes him, and he is carried home insensible, helpless, and incapable of uttering a word. No disease is more appalling in its suddenness than paralysis, and it should be matter for thankfulness to Londoners that less than 10,000 persons out of more than four millions were so seized during the year 1893. Let those that were spared then give freely to relieve those stricken with so distressing a malady.

Diagrams of Diseases.

### HOW DISEASE IS DISTRIBUTED THROUGHOUT LONDON.

HAVING shown the proportion which illness or disease bears to the population of London as a whole, it may be interesting to illustrate the extent to which men, women, and children resident in so vast a city are liable to illness in the course of each year. The series of diagrams which follow, and which are in scale with the population and the diseases to which they are liable, strikingly illustrate this point. It is astonishing to find, for example, that whereas the population includes upwards of 200,000 more women than men, considerably less than half a million women suffered from illness during the year 1893, although 560,000 men in round numbers were affected thereby. In other words, whereas one out of every two men required hospital treatment, only one in every three women received treatment at the hospitals during the year. The children occupy an intermediate position between the two sexes, for about one child in every two and a half of the whole population received hospital treatment.

We will now proceed to compare the whole population with that which was attacked by illness, grouping them according to sex and age.

### MEN OVER TWENTY.

The whole male population is represented by the figure in black on a stencilled ground, and the men who suffered from illness by a stencilled figure on a black ground. It will be seen that out of the whole popu-

Men, 1,136,884.

Total Men.

Men.

Men, 559,995.

Sick Men.

lation of 1,136,884 *men over twenty years of age*, 559,995 *men received treatment* at the London voluntary hospitals and dispensaries and the hospitals of the Metropolitan Asylums Board during 1893. This is a very large proportion, and shows that no less than one out of every two men found his way to one hospital at any rate during the year. This fact accounts, no doubt, in a very great measure for the circumstance that whereas the number of males born each year exceeds that of females, the tendency is for the males to gradually diminish in number until they fall considerably below the total of the female population. All population returns prove this fact, which may be accounted for by the circumstance that the male population has to bear a much greater strain and to incur risks to which women are not liable.

### WOMEN OVER TWENTY.

Of the whole population of London 1,340,288 persons were women, of whom 454,013 found their way to the hospitals for treatment in the course of the year. Having regard to the increasing number of hospitals for diseases peculiar to women, and to the dangers attending motherhood and the diseases peculiar to the sex, it is not a little remarkable that so few women should be attacked by illness necessitating hospital treatment. We venture to hope that now that the truth is placed upon record and proved by figures, the accuracy of which is beyond dispute, that a great wave of gratitude will animate the whole of the female population of our city, from the highest to the lowest. If this result should follow the publication of these facts, then a new day will dawn for the voluntary hospitals, for then the free offerings of those who have it in their power to contribute substantially to the support of these institutions must necessarily increase to an extent which has been notably absent during recent years. We ask every one of the women who read THE HOSPITAL to study the following diagrams which are drawn to scale, and show the proportion which disease bears to the whole female population in the metropolis at the present time, so far as hospital treatment is concerned. When we had

Women, 1,340,288.

Total Women.

Women.

Women, 454,015.

Sick Women.

worked out the figures we could scarcely realise that the results arrived at could be correct. A little reflection convinces us, however, that the intense sympathy which woman always excites in man is largely accountable for the misapprehension which has arisen as to the proportion and amount of sickness which affects men and women respectively in London at the present time. It must be understood that we have included all the cases treated in every kind of hospital, including those which are specially devoted to diseases of women. We confidently appeal to the women of London to arise in their strength on behalf of the hospitals, and to secure for them such a measure of pecuniary support in the way of annual subscriptions especially, that every reputable hospital will henceforth be placed on a secure financial basis, not only for the present year, but for all time.

Children, *i.e.*, Boys and Girls under Twenty.

Of the whole population of London in 1893, 1,829,239 consisted of boys and girls under twenty years of age. It is noteworthy, as showing the results of town life, its effects and character, that whereas the administra-

Children, 1,829,239.

Total Children.

Children.

Children. 706,177.

Sick Children.

tive County of London contains upwards of 200,000 more women than men, it contained nearly three-quarters of a million more children than men, and more than half-a-million more children than women in the year. Out of 1,829,239 boys and girls, 706,177 found their way to the hospitals for treatment during 1893. To enable our readers to grasp the amount of human misery represented by upwards of 700,000 children who attended the hospitals, suffering from every form of illness, accident, and disease to which flesh is heir, we will ask parents to recall what the illness of one child in one family means to the household. Think of it, mothers and fathers; realise all you have undergone in consequence of the anxiety which you have experienced when your little ones have been attacked by illness, and then attempt to reproduce that anxiety seven hundred thousand times, and you will at once grasp the volume of benefit, comfort, and succour which the hospitals of London have rendered to the parents of poor households everywhere throughout the great city of which we are proud to be citizens. If the accidents, and illness, and disease which afflict your fellow men and women fail to stir your hearts, surely the pathos of the thought of seven hundred thousand suffering children all seriously ill, many of them sick unto death, must stir your hearts to pity and move you to form a resolution to do something to help the hospitals. This volume of human misery affecting members of the population must specially appeal to human sympathy, if for no other reason, then because of the powerlessness of children in such circumstances to help themselves. We look forward to the time when Hospital Sunday will become a festival in every household, knitting all the population of this vast city together in one sustained effort to pour into the hospital exchequer all the funds which may be necessary to render it impossible that one little sufferer shall be without the promptest and fullest relief which human skill can devise. These tender little children exhibit a patience when ill which teaches their elders a lesson which none can fail to appreciate who have ever visited a children's ward and watched the little sufferers as they lie in their cots and bear their misfortunes with a fortitude worthy of the highest admiration and sympathy.

### Hospitals a Necessity.

As Hospital Sunday again comes round it is desirable that every one should recognise that this institution is not merely one more added to the multifarious modes of begging which exist on every side, but is the expression of a widespread conviction shared in by people of all parties and all creeds that the hospital system, as it now exists in London, is an absolute necessity, an integral part of the social economy of the metropolis.

No one must be content to think that either from his position or his isolation the hospitals are nothing to him. It is impossible to live in a great city without being affected and influenced for good or for ill by the system on which it is organised, by the methods and the means by which its social units are linked together, and made to influence each other; and one of the great organisations of London is its hospital system.

The important position occupied by the hospital system in the economies of a great city arises mainly from two great facts. On the one hand, there is the growing cost and complexity of treatment, the fact that every year while increase of knowledge points out better ways of curing disease, these ways demand greater space, more perfect appliances, and more complete alteration of the surroundings of the patient. On the other hand is the hard necessity imposed by increasing rent on all except the wealthy, of so economising the space in which they live, that, endurable as the conditions of life may be while health continues, they are absolutely destructive to those who suffer from disease.

The exigencies of modern life and the assumed necessity of all workers living within a short distance of great centres of exchange, have led to such an amount of over-crowding, that not only are the poor and the lower middle classes deprived, when ill, of the space and air necessary for their recovery, but they are also debarred from the benefits of modern science in the way of treatment.

However anxious a doctor may be to give his patients the full benefit of his knowledge, his difficulties are enormous when he practices among the poor. No sooner does he try to give them the benefit of his scientific training than he is met by the stubborn facts that his directions are not carried out, the food he orders either is not got or is not given, the nursing which is so sadly wanted, and on which may hinge the verdict of life or death, is performed as best it may be by a weary wife or ignorant neighbour, the air and space so necessary to recovery do not exist, and amid the disorder caused by sickness even common cleanliness cannot be procured. Amid such surroundings what is science to do?

Unless with unfeeling inhumanity we are prepared to say that the poor and unfortunate are beyond the pale and are to be debarred from the benefits of modern science, in the way of nursing and of treatment, when cast down by accident or worn out by disease, we must admit that treatment in hospitals is absolutely necessary, and that the provision of such hospitals is an essential part of the life of a great city.

Every day, and in every part of London, accidents are happening, varying, doubtless, in degree, but still incapacitating from work, and in a large proportion of them demanding prompt, efficient, and scientific treatment for the preservation, not only of life, but of wage-earning capacity. Our sympathies go out readily enough to men who one minute are in the enjoyment of perfect health, and the next, in consequence of some terrible catastrophe, are laid low, and for weeks, perhaps, may hover at death's door. Everyone admits the necessity of hospitals for the reception of such cases as these. People, however, are apt to make light of what they call trivial cases, and to forget that to the worker no injury is trivial. A small cut upon the finger, ill-treated, and rendering the hand less clever; a sprain, thought little of, but leaving a weakness and a limp; a bit of cinder or of steel in the eye, not immediately removed, and thus producing defective vision—small things in themselves—diminish a man's power of getting work, deteriorate his wage-earning power, and lessen his ability to support his family. And one of the most important benefits conferred on London by its hospitals is that they stand with open doors and with ever ready appliances, not only to give succour in the grave accidents which occur occasionally, but to give first aid and skilled assistance to that ever flowing stream of minor casualties which without such aid tend to impair the earning power of working people and to drive them towards the poorhouse.

To understand the necessity which is laid upon London rs of liberally supporting the London hospitals we must think of concrete examples. Abstract theories as to the importance of self-help or as to the fear of evils which may result from charity, all fall to the ground at the sight of an accident or in presence of a case of acute disease dying from the impossibility of proper treatment in his home, yet capable of restoration to usefulness if dealt with in a hospital.

At any minute we may be in the presence of an accident. A slip at a crossing, a passing cab, and the and the thing is done. There is the man suffering from some frightful injury, perhaps unconscious, unable to give name or address. As a community we have to answer the question—what is to be done? And without hesitation, from the ragamuffin who runs round for the ambulance to the policeman who takes charge of the case, everyone answers, "take him to the hospital." It is the only thing to do, and London *must* support its hospitals.

Not so long ago a scaffolding, on which a number of decorators were engaged, fell down among the loungers in a West-end thoroughfare, who in a moment found themselves in the presence of the dead and the dying. Again, the only possible thing to do was to take them to the hospital. To attempt to deal with cases of this sort in any other way is absolutely out of the question. Take the case of a railway smash, or one of these poor fellows precipitated from a scaffold, or the far commoner case of a compound fracture, caused by being run over in the streets. The patient is helpless, maybe unconscious, and nearly dead. Everything depends on immediate treatment. To hurry such a case off to a distant home where all is unprepared is practically to kill him; it is in hospitals alone that such cases can obtain the immediate assistance they require.

At the hospital everything is ready; there is plenty of help, and that help is trained. In a few minutes the patient is in bed, his circulation is being restored by hot bottles placed around him, his hæmorrhage has been stayed, his wounds are dressed, and Nature, that wonderful power of recovery from injury which every animal possesses in more or less degree, at least has a chance of helping the sufferer back to life.

How different in the tenement. The crooked staircase, in the ascent of which every injury is intensified and every pain made worse; the empty house, guarded may be by one or two children, their mother and their elders being out at work; the empty grate; the badly-placed bed; the weary waiting while the doctor is being sent for; his helplessness when he arrives for want of skilled assistance; the shivering, sinking patient, wanting warmth while the frightened children upset the kettle in trying to make it boil; the futile efforts to stop the bleeding; the doctor working by the light of a dip candle, without sponges or proper appliances of any sort; these are things once seen to be remembered. As a fact, they do not happen often, because of the existence of our hospital system; but that they would and must happen unless our hospitals were properly supported is obvious when we consider for a moment the sort of dwellings in which a large proportion of the people of London live.

Nearly half the entire population of London lives in tenements consisting of not more than four rooms each. According to the report of the medical officer to the County Council more than 600,000 of such tenements exist, of which a very large proportion contain only two, and a very considerable proportion only one room each. In such dwellings it is out of the question to treat cases of serious accident or disease. Hospitals of some sort or another are an absolute necessity; whether they are supported by charity or by the rates they must continue, and no one who has investigated the matter can hesitate for a moment as to the enormous advantage of keeping the hospitals on a voluntary charitable basis rather than handing them over to the management of rate-paid officials.

"JUST BEFORE BEDTIME."

QUEEN VICTORIA WARD, THE LONDON HOSPITAL, E.

### WAYS AND MEANS: A WORD TO LIVING LONDONERS.

### THE COST OF THE WORK DONE.

DURING December and January last articles and correspondence appeared in the *Times* under the heading "Is Charity in extremis?" which dealt with the relative financial difficulties of hospitals and other charities in recent years. We have no space to go into the matter on the present occasion, but the fact is worthy of note in connection with this branch of the subject. In the year 1893 the total expenditure of the London voluntary hospitals, including St. Bartholomew's, Guy's, and St. Thomas's, and the metropolitan dispensaries was £790,229. In other words, it required *upwards of three-quarters of a million of money* to defray the cost of providing adequate hospital treatment for about 1,696,276 patients. We have excluded from this calculation 20,911 fever cases treated at the hospitals of the Metropolitan Asylums Board and the cost of such treatment. *The total income of the London voluntary hospitals* and dispensaries during 1893 was £766,245, which was derived from the following sources:—

| | |
|---|---|
| Charitable or voluntary contributions... | £304,259 |
| Income from invested property... ... | 255,427 |
| Legacies ... ... ... ... ... | 152,106 |
| Patients' payments ... ... ... | 54,453 |

In the above figures the income and expenditure of St. Bartholomew's and St. Thomas's Hospitals have been confined to that portion of their revenue and expenditure which was applicable to hospital purposes.

### HOW THE MONEY IS PROVIDED.

Let us now consider where the money came from to pay the cost of the hospital relief given to the inhabitants of London by these voluntary institutions. In order to bring home the facts to the meanest comprehension, we have prepared diagrams each representing a hand and a coin, which have been drawn to scale, and show exactly the proportion of every sovereign which was contributed by the living, who received all the benefits, and by deceased benefactors, many of whom took an active part in the management of hospitals during their lifetime, and whose benefactions have enabled them to meet the ever increasing needs of upwards of four millions of people. With a view to clearness and ready comprehension, the diagrams have been drawn so as to represent the exact proportion given of every sovereign expended, by (*a*) the living, (*b*) the dead, and (*c*) the patients themselves. Of every sovereign expended 10s. 6d., or *more than one-half, is derived from legacies and the interest upon gifts* of deceased benefactors which have been invested in approved securities; 7s. 6d. out of every sovereign has been given in charity by the present population of London, that is, the living for whose benefit the hospitals exist; and 1s. 6d. out of every sovereign has been contributed by the patients treated in the hospitals. *The balance of* 6d. *in the pound represents the annual deficiency*, which amounted in 1893 to £23,984. In other words, *if the contributions to the hospitals on Hospital Sunday in London can be raised from* £45,000 *to* £70,000 *the existing voluntary hospitals will be able to fulfil all the work cast upon them and to pay their way*. We may add that *the black hand* and the coin held by it represent the dead hand, *i.e.*, the contributions from those now dead, *the white hand* represents the charitable contributions of the living, and the smallest coin the amount derived from patients' payments.

### THE MEANING OF THE DIAGRAMS.

We desire to direct the attention of all classes in London to the foregoing figures. They show that, whereas the people of London resort to the voluntary hospitals in greater numbers than the population of any other city in the United Kingdom, they miserably fail to recognise their duty to the hospitals and to show their sense of the benefits they have received from these institutions. Every provincial city of importance takes the deepest pride in its hospitals

**The Living gave**

**7s. 6d. in the £1.**

**The Living, i.e., the present inhabitants, only give 7s. 6d of each £1 expended by the Voluntary Hospitals.**

**The Dead Hand gave**

**10s. 6d. in the £1.**

**The Dead Hand gives 10s. 6d. out of every £1 expended by the Hospitals.**

and provides them with adequate funds. It follows that in provincial cities, instead of the hospitals being starved and impoverished for want of liberal support at the hands of the living, who are continually demanding and always receiving increasing benefits from these institutions, all classes of the population combine to provide the necessary funds in adequate proportions. In London almost the exact opposite is the case. The living show their appreciation of the hospitals by demanding greater value in relief every year at the hands of the hospitals, whilst they fail to contribute anything like a proportionate sum in payment for the benefits they receive. Is there one intelligent citizen of London who will not blush to find that the population of this vast city in the year 1893 contributed barely more than one-third of the sum which was expended in affording hospital relief to the citizens as a whole?

Had it not been for the contributions of the dead hand, which amount at the present time to 10s. 6d. out of every pound, more than half the hospitals must have been closed for want of funds. We appeal to the people of London to exhibit more self-respect

**Patients' Payments supply 1s. 6d. of each £1 expended by the Voluntary Hospitals.**

and a determination to henceforth follow the noble example set them in this matter by their forebears and sires.

But this is not the whole case, for if the 1s. 6d. in the pound derived from patients' payments be credited to the living, *i.e.*, to the present inhabitants of London, then the sum yielded only amounts to 9s. in the pound, as compared with a contribution of 10s. 6d. in the pound from those now deceased who cannot in any sense benefit by the existence of our voluntary hospitals.

We hope that the Press will make these facts widely known, and that the living will arouse themselves sufficiently and so increase their contributions on Hospital Sunday this year that the charitable contributions, combined with patients' payments, will at least amount to 10s. 6d. in every sovereign. Any less result would be discreditable indeed to the present inhabitants of London. Although it is true that the total deficiency of all the London Hospitals in 1893 represents but sixpence in the pound, still there are hospitals like the Westminster, Charing Cross, and King's College, which are urgently in need of large sums to defray current expenses, whilst the district of South-east London, containing 1½ millions of the population, has no adequate hospital accommodation at all, a fact which is making itself felt with increasing urgency at the present time. An extra 1s. 6d. in the pound from the living would put all the existing hospitals in funds. It would also provide money enough to maintain a large general hospital at Camberwell, where it is sadly needed, as the report of the Lords' Committee abundantly proved.

### Raising the Wind.

In view of the enormous annual expenditure required to maintain the hospitals of London in full efficiency and of the fact that no public funds are available for the purpose, it is of the greatest importance that every possible voluntary agency should be pressed into the service. The days are gone by when a hospital could safely trust to legacies and annual subscriptions, paid almost without asking, in regular course. The distribution of wealth is different, the part taken in public life by different classes is different, and the importance of the earnings of the many compared with the interest of the few is very different from what it was years ago. An entirely new middle class has sprung into existence, recruited from the *élite* of the workers, who have not yet accepted the easy and mechanical method of charity by subscription list, and hospitals must suit their methods of raising money to the altered conditions of the time. Money must now be collected not from the few, but from the many, and everyone must be interested in the process. In America and in many of our provincial towns it is fully recognised that man is swayed by his fellows, and that appeals which fall flat when made to isolated individuals are fertile in their productiveness when addressed to bodies of men who are accustomed in their daily lives to work together. Men's organisations are connected with their religions, their trades, or their amusements. All of these ought to be induced to show interest in the hospitals. Football and cricket clubs, athletic societies, musical and theatrical associations, all have a balance of some sort or another at the end of the year's operations, which might as well be presented to the hospital. On every wage-day in every mill a penny might be collected at the time of payment, and experience teaches that where this is regularly done it is not missed, and that contributions so made amount in some cases to as much as all the subscriptions collected in the ordinary way. Every Church of whatever denomination can well afford to pass the plate for the hospitals. The religious life of the country is organised by congregation, and each congregation, accustomed as it is to give to various and strange objects, according to the leanings and sympathies of its priest or minister, may well ask to have one Sunday set aside on which to give to the hospitals in its midst. But all these things require organisation, and require popular interest to be excited. In regard to the congregations we already have the organisation in the Hospital Sunday; in regard to the popular interest the difficulty is considerable. All successful institutions tend to suffer from a general faith in their prosperity. People will not trouble to help what they think is in a flourishing state. It remains, then, for us very strongly to insist that whatever the collections made on Hospital Sundays may have been, they ought to be greater still. Times alter every year, and every year more dependence has to be placed upon the general, promiscuous, widespread giving of the public, in addition to the more or less stationary value of the subscription list. For this purpose every means by which a general interest in the subject is of importance. To get the thing talked about, to make people think, to induce them to realise the necessity of hospitals to the community, and their constant want of funds, is half the battle. An American work on hospitals lately published says that, after all, "it is by talking, talking, talking," that general interest in a hospital is obtained, and in regard to Hospital Sunday we may transpose an old adage by saying, he gives twice who makes his neighbour give.

### Metropolitan Hospital Sunday Fund, 1895.

### A YEAR'S WORK IN THE HOSPITALS AND MEDICAL CHARITIES OF LONDON.

### NEWINGTON AND SOUTH DISTRICT.

Comprising Battersea, Wandsworth, Tooting, Balham, Streatham, Brixton, Lambeth, Newington, Southwark, Bermondsey, Camberwell, Greenwich, Deptford, Lewisham, Blackheath, Woolwich, &c.

| No. of Beds. | No. of Beds Daily Occupied. | HOSPITALS. | In-patients. | Out-patients. | Total Expenditure. | Income. | | | Total Income. | Legacies not included in previous column. |
|---|---|---|---|---|---|---|---|---|---|---|
| | | | | | | Charitable. | Proprietary. | Patients' Payments. | | |
| | | | | | £ | £ | £ | £ | £ | £ |
| 600 | 411 | Guy's ... ... ... ... ... | 6,160 | 56,832 | 41,659 | 9,476 | 23,300 | 4,386 | 37,162 | ... |
| 23 | 20 | Miller ... ... ... ... ... | 283 | 15,707 | 3,551 | 1,966 | 399 | ... | 2,365 | 290 |
| 253 | 210 | Seamen's ... ... ... ... ... | 2,404 | 15,023 | 14,918 | 8,389 | 4,009 | 77 | 12,475 | 1,050 |
| 66 | 56 | Evelina, for Children ... ... | 743 | 8,144 | 6,395 | 4,211 | 540 | 90 | 4,841 | 50 |
| 51 | 40 | Home for Sick Children ... ... | 191 | 1,865 | 1,585 | 1,067 | 56 | 318 | 1,441 | 7,141 |
| 24 | 21 | General Lying-In ... ... ... | 535 | 1,624 | 3,440 | 696 | 2,523 | ... | 3,219 | ... |
| 36 | 13 | Clapham Maternity and Dispensary | 302 | 4,930 | 1,385 | 310 | 493 | 512 | 1,315 | ... |
| 51 | 49 | Royal, for Children and Women ... | 591 | 8,067 | 3,940 | 2,153 | 809 | 212 | 3,174 | ... |
| 40 | 20 | Royal Eye ... ... ... ... ... | 490 | 15,337 | 5,702 | 2,114 | 149 | 502 | 2,765 | 237 |
| 10 | 1 | Hospital for Diseases of the Skin ... | 14 | 4,995 | 1,110 | 219 | 152 | 560 | 931 | ... |
| 352 | 204 | Metropolitan Convalescent ... ... | 3,125 | ... | 6,881 | 5,093 | 605 | ... | 5,698 | 101 |
| 10 | 4 | Phillips' Memorial Homœopathic ... | 75 | 349 | 573 | 425 | 10 | 187 | 622 | ... |
| 10 | 7 | Eltham Cottage ... ... ... | 70 | 10 | 406 | 328 | 10 | 105 | 443 | 100 |
| 18 | 10 | Beckenham Cottage ... ... ... | 100 | ... | 635 | 549 | 23 | 89 | 661 | ... |
| 20 | 10 | Blackheath Cottage ... ... ... | 127 | 965 | 796 | 841 | 32 | 111 | 984 | ... |
| 20 | 12 | Bromley Cottage ... ... ... | 189 | ... | 964 | 1,879 | 73 | 114 | 2,066 | ... |
| 14 | 9 | Chislehurst, &c., Cottage ... ... | 124 | ... | 618 | 752 | 2 | 132 | 886 | ... |
| 12 | 8 | Sidcup Cottage ... ... ... | 100 | 50 | 484 | 354 | 11 | 114 | 479 | ... |
| 11 | 7 | Woolwich Cottage ... ... ... | 78 | 30 | 400 | 316 | 10 | 69 | 395 | ... |
| 1,621 | 1,112 | | 15,701 | 133,928 | 95,442 | 41,138 | 33,206 | 7,578 | 81,922 | 8,969 |
| | | DISPENSARIES. | | | | | | | | |
| ... | ... | Battersea Provident ... ... ... | ... | 18,794 | 2,696 | 86 | 62 | 2,514 | 2,662 | ... |
| ... | ... | Brixton, &c. ... ... ... ... | ... | 15,207 | 786 | 688 | 21 | 94 | 803 | ... |
| ... | ... | Camberwell Provident ... ... | ... | 11,526 | 2,073 | 433 | 127 | 1,437 | 1,997 | ... |
| ... | ... | Clapham ... ... ... ... ... | ... | 1,128 | 366 | 324 | ... | 91 | 415 | 100 |
| ... | ... | Deptford Medical Mission ... ... | ... | 2,882 | 534 | 496 | 113 | 52 | 661 | ... |
| ... | ... | East Dulwich Provident ... ... | ... | 1,053 | 630 | 92 | 1 | 539 | 632 | ... |
| ... | ... | Forest Hill ... ... ... ... | ... | 1,973 | 685 | 289 | ... | 433 | 722 | ... |
| ... | ... | Royal South London... ... ... | ... | 4,242 | 695 | 594 | 112 | ... | 706 | ... |
| .. | ... | South Lambeth, &c.... ... ... | ... | 2,230 | 571 | 380 | 16 | 188 | 584 | ... |
| ... | ... | Walworth Provident ... ... | ... | 774 | 255 | 12 | 33 | 125 | 170 | ... |
| ... | ... | Wandsworth Common ... ... | ... | 789 | 191 | 52 | ... | 140 | 192 | ... |
| 1,621 | 1,112 | | 15,701 | 194,526 | 104,924 | 44,584 | 33,691 | 13,191 | 91,466 | 9,069 |

### CITY AND EAST CENTRAL DISTRICT.

Comprising the City, St. Luke's, Shoreditch, Finsbury, and Clerkenwell.

| No. of Beds. | No. of Beds Daily Occupied. | HOSPITALS. | In-patients. | Out-patients. | Total Expenditure. | Income. | | | Total Income. | Legacies not included in previous column. |
|---|---|---|---|---|---|---|---|---|---|---|
| | | | | | | Charitable. | Proprietary. | Patients' Payments. | | |
| | | | | | £ | £ | £ | £ | £ | £ |
| 62 | 56 | Metropolitan ... ... ... ... | 781 | 16,033 | 8,877 | 5,309 | 430 | 975 | 6,714 | 817 |
| 160 | 105 | Royal Free ... ... ... ... | 1,460 | 27,680 | 9,760 | 4,000 | 1,253 | ... | 5,353 | 3,142 |
| 80 | 34 | Royal, for Diseases of the Chest ... | 477 | 5,474 | 8,249 | 5,948 | 121 | ... | 6,069 | 623 |
| 57 | 52 | North-Eastern, for Children ... | 701 | 13,124 | 6,009 | 4,444 | 564 | 637 | 5,645 | 30 |
| 36 | 21 | City of London Lying-In ... ... | 492 | 1,697 | 3,852 | 774 | 3,459 | ... | 4,233 | 270 |
| 34 | 7 | St. Mark's, for Fistula ... ... | 55 | 731 | 1,808 | 848 | 975 | ... | 1,823 | 3,723 |
| 100 | 85 | Royal London Ophthalmic ... ... | 1,980 | 24,511 | 7,006 | 2,833 | 932 | ... | 3,765 | 3,863 |
| 30 | 25 | City Orthopædic ... ... ... | 175 | 2,078 | 1,261 | 1,233 | 32 | ... | 1,265 | ... |
| 17 | 12 | Central London Throat and Ear ... | 258 | 7,393 | 2,309 | 545 | 155 | 1,313 | 2,013 | 380 |
| 566 | 417 | | 6,379 | 98,721 | 49,131 | 25,934 | 7,921 | 2,925 | 36,780 | 12,848 |
| | | DISPENSARIES. | | | | | | | | |
| ... | ... | City ... ... ... ... ... | ... | 4,890 | 1,300 | 916 | 182 | ... | 1,098 | ... |
| ... | ... | City of London and East London ... | ... | 10,947 | 1,074 | 117 | 37 | 1,007 | 1,161 | ... |
| ... | ... | Farringdon General ... ... ... | ... | 6,042 | 625 | 283 | ... | 315 | 598 | .. |
| ... | ... | Finsbury ... ... ... ... ... | ... | 12,590 | 843 | 547 | 144 | 247 | 938 | ... |
| ... | .. | Metropolitan ... ... ... ... | ... | 7,380 | 884 | 549 | 118 | 313 | 980 | ... |
| ... | ... | Royal General ... ... ... ... | ... | 3,630 | 903 | 392 | 490 | 92 | 974 | ... |
| 566 | 417 | | 6,379 | 144,200 | 54,760 | 28,738 | 8,892 | 4,899 | 42,529 | 12,848 |

### ST. MARYLEBONE AND WEST CENTRAL DISTRICT.

Comprising St. Marylebone, St. John's Wood, Bloomsbury, Holborn, &c.

| No. of Beds. | No. of Beds Daily Occupied. | HOSPITALS. | In-patients. | Out-patients. | Total Expenditure. | Income. | | | Total Income. | Legacies not included in previous column. |
|---|---|---|---|---|---|---|---|---|---|---|
| | | | | | | Charitable. | Proprietary. | Patients' Payments. | | |
| | | | | | £ | £ | £ | £ | £ | £ |
| 70 | 53 | French ... ... ... ... ... | 825 | 6,179 | 4,721 | 3,907 | 48 | ... | 3,955 | 135 |
| 20 | 12 | Italian ... ... ... ... ... | 211 | 4,583 | 889 | 709 | 99 | ... | 808 | ... |
| 40 | 36 | London Homœopathic ... ... | 496 | 11,376 | 5,397 | 1,600 | 3,039 | 482 | 5,121 | 1,178 |
| 51 | 43 | SS. John and Elizabeth ... ... | 94 | ... | 1,632 | 834 | 828 | ... | 1,662 | 20 |
| 321 | 269 | The Middlesex ... ... ... | 3,182 | 38,122 | 31,001 | 8,543 | 9,021 | ... | 17,564 | 15,420 |
| 68 | 65 | Alexandra for Children ... ... | 166 | 244 | 2,412 | 1,705 | 118 | 437 | 2,260 | 90 |
| 30 | 29 | Hospital for Incurable Children ... | 37 | ... | 1,178 | 556 | 23 | 393 | 972 | 986 |
| 230 | 142 | Hospital for Sick Children ... ... | 1,9[illegible]2 | 27,334 | 16,065 | 9,910 | 3,207 | ... | 13,117 | 7,828 |
| 16 | 9 | British Lying-In ... ... ... | 193 | 290 | 1,707 | 438 | 1,204 | 10 | 1,652 | ... |
| 58 | 41 | Queen Charlotte's Lying-In... ... | 1,079 | 1,318 | 4,784 | 2,762 | 373 | 75 | 3,210 | 528 |
| 42 | 35 | New Hospital for Women ... ... | 494 | 11,905 | 4,000 | 2,157 | 251 | 1,277 | 3,685 | 313 |
| 53 | 50 | Samaritan Free ... ... ... | 575 | 8,910 | 7,193 | 5,738 | 177 | ... | 5,915 | 1,619 |
| 180 | 172 | National for Paralysed, &c.... ... | 927 | 5,000 | 13,595 | 4,569 | 1,490 | 1,976 | 8,035 | 1,973 |
| 25 | 19 | Hospital for Epilepsy, &c. ... ... | 98 | 720 | 1,898 | 920 | 67 | 656 | 1,643 | ... |
| 50 | 38 | West End for Epilepsy, &c.... ... | 283 | 2,150 | 2,324 | 1,293 | 260 | 429 | 1,982 | ... |
| 13 | 7 | Central London Ophthalmic ... | 230 | 9,890 | 1,084 | 639 | 88 | 401 | 1,128 | ... |
| 15 | 6 | Western Ophthalmic... ... ... | 148 | 5,110 | 688 | 497 | 134 | 6 | 637 | ... |
| 60 | 57 | National Orthopædic ... ... | 216 | 748 | 2,264 | 824 | ... | 1,292 | 2,116 | ... |
| 25 | 15 | Establishment for Gentlewomen ... | 127 | ... | 2,125 | 715 | 131 | 966 | 1,812 | 225 |
| ... | ... | National Dental ... ... ... | ... | 31,851 | 1,516 | 1,340 | ... | 200 | 1,540 | ... |
| 1,367 | 1,098 | | 11,343 | 165,530 | 106,473 | 49,656 | 20,558 | 8,600 | 78,814 | 30,315 |
| | | DISPENSARIES. | | | | | | | | |
| ... | ... | Bloomsbury Provident ... ... | ... | 479 | 269 | 11 | ... | 213 | 224 | ... |
| ... | ... | London Medical Mission ... ... | 155 | 2,390 | 998 | 737 | 73 | 129 | 939 | ... |
| ... | ... | Portland Town ... ... ... | ... | 2,434 | 171 | 149 | 5 | 13 | 167 | ... |
| ... | ... | Portobello Road ... ... ... | ... | 149 | 75 | 48 | 4 | 27 | 79 | ... |
| ... | ... | St. John's Wood Provident... ... | ... | 5,115 | 532 | 261 | 32 | 275 | 568 | ... |
| ... | ... | St. Marylebone General ... ... | ... | 4,200 | 945 | 306 | 126 | 319 | 751 | 200 |
| ... | ... | Western General ... ... ... | ... | 17,070 | 1,393 | 1,110 | 31 | ... | 1,141 | 250 |
| 1,367 | 1,098 | | 11,498 | 197,367 | 110,856 | 52,278 | 20,829 | 9,576 | 82,683 | 30,765 |

### KENSINGTON AND WEST DISTRICT.

Comprising Kensington, Paddington, Bayswater, Kilburn, Chelsea, Brompton, Fulham, Hammersmith, Chiswick, Brentford, Acton, Ealing, &c.

| | | HOSPITALS. | | | £ | £ | £ | £ | £ | £ |
|---|---|---|---|---|---|---|---|---|---|---|
| 12 | 10 | Queen's Jubilee ... ... ... | 179 | 9,749 | 1,677 | 1,698 | 13 | ... | 1,711 | ... |
| 351 | 328 | St. George's ... ... ... ... | 3,968 | 31,164 | 38,757 | 10,104 | 15,374 | ... | 25,478 | 18,316 |
| 281 | 250 | St. Mary's ... ... ... ... | 3,874 | 35,487 | 22,060 | 13,257 | 2,354 | ... | 15,611 | 3,620 |
| 101 | 92 | West London... ... ... ... | 1,441 | 31,933 | 6,854 | 5,120 | 228 | ... | 5,348 | 1,290 |
| 321 | 276 | Hospital for Consumption ... ... | 1,543 | 13,750 | 28,953 | 12,623 | 8,339 | ... | 20,962 | 3,633 |
| 24 | 22 | Belgrave, for Children ... ... | 243 | 2,934 | 1,547 | 1,663 | 77 | ... | 1,740 | ... |
| 50 | 49 | Cheyne, for Sick & Incurable Children | 75 | ... | 2,478 | 2,036 | 205 | 504 | 2,745 | ... |
| 23 | 18 | Paddington Green, for Children ... | 175 | 9,123 | 1,846 | 1,512 | 165 | 157 | 1,834 | 985 |
| 120 | 112 | Victoria, for Children ... ... | 1,734 | 20,732 | 8,370 | 6,417 | 823 | 489 | 7,729 | 551 |
| 42 | 25 | Chelsea, for Women ... ... | 347 | 2,510 | 5,515 | 4,581 | 62 | 498 | 5,441 | 200 |
| 105 | 82 | Cancer ... ... ... ... ... | 787 | 1,513 | 11,586 | 5,162 | 2,287 | ... | 7,449 | 6,136 |
| 135 | 97 | Female Lock ... ... ... ... | 729 | ... | 3,838 | 1,892 | ... | 1,919 | 3,811 | 200 |
| 22 | 19 | Male Lock ... ... ... ... | 306 | 4,892 | 1,827 | 120 | ... | 1,951 | 2,071 | ... |
| 16 | 10 | Epsom and Ewell Cottage ... . | 115 | ... | 890 | 628 | 4 | 206 | 838 | ... |
| 18 | 14 | Reigate and Redhill Cottage .. | 207 | ... | 736 | 536 | 56 | 96 | 688 | ... |
| 11 | 6 | Wimbledon Cottage ... ... ... | 111 | ... | 548 | 358 | 5 | 84 | 447 | ... |
| 1,662 | 1,436 | | 15,833 | 163,787 | 137,492 | 67,707 | 29,992 | 5,904 | 103,603 | 34,931 |
| | | DISPENSARIES. | | | | | | | | |
| ... | ... | Brompton Provident... ... ... | ... | 1,397 | 506 | 165 | 82 | 262 | 509 | ... |
| ... | ... | Chelsea, &c. ... ... ... ... | ... | 4,787 | 654 | 447 | 185 | ... | 632 | ... |
| ... | ... | Chelsea Provident ... ... ... | ... | 681 | 262 | 34 | ... | 172 | 206 | ... |
| ... | ... | Kensal Town Provident ... ... | ... | 761 | 349 | 35 | 52 | 254 | 341 | ... |
| ... | ... | Kensington ... ... ... ... | ... | 4,302 | 667 | 520 | 53 | ... | 573 | 180 |
| ... | ... | Kilburn, Maida Vale ... ... | ... | 3,261 | 570 | 454 | 71 | ... | 525 | ... |
| ... | ... | Kilburn Provident ... ... ... | ... | 6,018 | 1,068 | 63 | 10 | 995 | 1,068 | ... |
| ... | ... | Notting Hill Provident ... ... | ... | 1,068 | 207 | 96 | ... | 80 | 176 | ... |
| ... | ... | Paddington Provident ... ... | ... | 3,846 | 551 | 144 | 25 | 357 | 526 | ... |
| ... | ... | Pimlico Provident ... ... ... | ... | 1,922 | 526 | 17 | ... | 504 | 521 | ... |
| ... | ... | Royal Pimlico Provident ... ... | ... | 6,414 | 876 | 334 | 17 | 547 | 898 | 100 |
| ... | ... | Westbourne Provident ... ... | ... | 1,440 | 403 | 55 | 42 | 302 | 399 | ... |
| 1,662 | 1,436 | | 15,833 | 199,484 | 144,131 | 70,071 | 30,529 | 9,377 | 109,977 | 35,211 |

### ISLINGTON AND NORTH-WEST DISTRICT.

Comprising Islington, Holloway, Highbury, Hampstead, Highgate, St. Pancras, Stoke Newington, Tottenham, &c.

| No. of Beds. | No. of Beds Daily Occupied. | Hospitals. | In-patients. | Out-patients. | Total Expenditure. | Income. Charitable. | Income. Proprietary. | Income. Patients' Payments. | Total Income. | Legacies not included in previous column. |
|---|---|---|---|---|---|---|---|---|---|---|
| | | | | | £ | £ | £ | £ | £ | £ |
| 112 | 86 | Great Northern Central | 1,201 | 23,154 | 8,157 | 6,396 | 1,110 | 85 | 7,591 | 132 |
| 28 | 13 | Hampstead Hospital | 221 | 177 | 3,004 | 1,917 | 952 | 409 | 3,278 | ... |
| 120 | 67 | London Temperance | 1,044 | 11,120 | 10,399 | 5,406 | 2,248 | 220 | 7,874 | 153 |
| 53 | 46 | North West London | 530 | 16,855 | 4,112 | 4,228 | 135 | 44 | 4,407 | ... |
| 103 | 64 | Tottenham | 934 | 13,820 | 5,618 | 3,539 | 164 | 980 | 4,683 | 1,139 |
| 208 | 181 | University College | 3,305 | 43,571 | 19,531 | 12,366 | 45 | 5,028 | 17,439 | 4,458 |
| 54 | 48 | North London Consumption | 413 | 3,052 | 5,707 | 5,833 | 117 | 34 | 5,984 | 500 |
| 170 | 56 | London Fever | 362 | ... | 13,044 | 6,843 | 2,015 | 1,681 | 10,539 | 743 |
| 28 | 18 | Invalid Asylum | 197 | ... | 899 | 480 | 130 | 186 | 796 | ... |
| 16 | 12 | Children's Home Hospital, Barnet | 62 | ... | 523 | 354 | 17 | 151 | 522 | ... |
| 9 | 6 | Enfield Cottage | 68 | ... | 397 | 334 | 17 | 32 | 383 | ... |
| 801 | 597 | | 8,607 | 111,749 | 71,391 | 47,696 | 6,950 | 8,850 | 63,496 | 7,125 |
| | | DISPENSARIES. | | | | | | | | |
| ... | ... | Camden Provident | ... | 914 | 284 | 15 | ... | 228 | 243 | ... |
| ... | ... | Hampstead Provident | ... | 10,956 | 1,017 | 289 | 57 | 660 | 1,006 | ... |
| ... | ... | Holloway and North Islington | ... | 3,838 | 936 | 344 | 57 | 346 | 747 | 50 |
| ... | ... | Islington | ... | 10,142 | 859 | 289 | 17 | 510 | 816 | ... |
| ... | ... | St. Pancras and Northern | ... | 1,618 | 539 | 299 | 98 | 81 | 478 | ... |
| ... | ... | Stamford Hill, &c. | ... | 4,649 | 609 | 467 | 136 | ... | 603 | 25 |
| 801 | 597 | | 8,067 | 143,866 | 75,635 | 49,399 | 7,315 | 10,675 | 67,389 | 7,200 |

### WESTMINSTER DISTRICT.

Comprising Westminster City and Liberties.

| No. of Beds. | No. of Beds Daily Occupied. | Hospitals. | In-patients. | Out-patients. | Total Expenditure. | Income. Charitable. | Income. Proprietary. | Income. Patients' Payments. | Total Income. | Legacies not included in previous column. |
|---|---|---|---|---|---|---|---|---|---|---|
| | | | | | £ | £ | £ | £ | £ | £ |
| 175 | 140 | Charing Cross | 2,050 | 23,696 | 15,270 | 9,015 | 2,783 | ... | 11,798 | 3,085 |
| 220 | 176 | King's College | 2,304 | 22,646 | 20,109 | 12,148 | 2,003 | 98 | 14,249 | 6,239 |
| 205 | 181 | Westminster | 2,934 | 24,559 | 15,805 | 4,384 | 3,068 | ... | 7,452 | 2,204 |
| 134 | 116 | Ventnor, for Consumption | 793 | ... | 11,245 | 6,294 | 1,780 | 3,353 | 11,427 | 842 |
| 18 | 11 | Grosvenor, for Women and Children | 135 | 2,000 | 1,582 | 904 | 9 | 330 | 1,243 | ... |
| 65 | 43 | Hospital for Women | 566 | 5,068 | 6,022 | 2,303 | 250 | 500 | 3,053 | 3,037 |
| 25 | 21 | National, for Diseases of Heart, &c. | 132 | 1,862 | 2,063 | 1,462 | 22 | 352 | 1,836 | 150 |
| 30 | 19 | Royal Westminster Ophthalmic | 423 | 9,679 | 2,041 | 1,800 | 365 | 43 | 2,208 | 168 |
| 50 | 49 | Royal Orthopædic | 126 | 847 | 1,999 | 1,269 | 520 | 129 | 1,918 | ... |
| 26 | 22 | Hospital for Diseases of the Throat | 610 | 7,699 | 3,324 | 801 | 225 | 2,860 | 3,886 | 1,000 |
| 9 | 6 | Royal Ear | 204 | 2,146 | 710 | 129 | 4 | 587 | 720 | ... |
| ... | ... | Dental | ... | 44,810 | 2,180 | 1,703 | 264 | 283 | 2,250 | ... |
| 20 | 13 | Gordon, for Fistula | 211 | 740 | 1,402 | 584 | ... | 756 | 1,340 | ... |
| 27 | 17 | St. Peter's, for Stone | 445 | 4,722 | 3,895 | 826 | 523 | 2,347 | 3,696 | ... |
| 1,004 | 805 | | 10,933 | 150,474 | 87,647 | 43,622 | 11,816 | 11,638 | 67,076 | 16,625 |
| | | DISPENSARIES. | | | | | | | | |
| ... | ... | Public | ... | 3,417 | 696 | 468 | 146 | ... | 614 | ... |
| ... | ... | St. George and St. James | ... | 4,053 | 539 | 274 | 13 | 24 | 311 | ... |
| ... | ... | St. George, Hanover Square | ... | 1,982 | 574 | 366 | 20 | 169 | 555 | ... |
| ... | ... | Western | ... | 3,472 | 1,331 | 396 | 395 | 560 | 1,351 | ... |
| ... | ... | Westminster General | ... | 4,209 | 1,099 | 519 | 148 | 39 | 706 | 358 |
| 1,004 | 805 | | 10,933 | 167,607 | 91,886 | 45,645 | 12,538 | 12,430 | 70,613 | 16,983 |

### STRATFORD AND EAST-END DISTRICT.

Comprising Bethnal Green, Tower Hamlets, West Ham, Whitechapel, Hackney, Stepney, Limehouse, Poplar, and the East

| No. of Beds. | No. of Beds Daily Occupied. | Hospitals. | In-patients. | Out-patients. | Total Expenditure. | Income. Charitable. | Income. Proprietary. | Income. Patients' Payments. | Total Income. | Legacies not included in previous column. |
|---|---|---|---|---|---|---|---|---|---|---|
| | | | | | £ | £ | £ | £ | £ | £ |
| 120 | 98 | German | 1,156 | 21,312 | 9,285 | 5,159 | 2,257 | 188 | 7,604 | 47 |
| 776 | 625 | London | 9,089 | 128,315 | 64,975 | 28,333 | 22,549 | 400 | 51,282 | 18,339 |
| 64 | 34 | Poplar | 719 | 14,597 | 14,864 | 5,007 | 8,628 | ... | 13,635 | 153 |
| 39 | 24 | West Ham, &c. | 272 | 17,088 | 3,167 | 2,858 | 265 | ... | 3,123 | ... |
| 164 | 106 | City of London for Dis. of the Chest | 952 | 16,054 | 12,212 | 6,000 | 274 | ... | 6,274 | 3,183 |
| 102 | 87 | East London for Children | 1,424 | 30,454 | 7,394 | 6,916 | 937 | ... | 7,853 | 3,146 |
| 92 | 43 | Mrs. Gladstone's Home | 743 | ... | 1,121 | 585 | 377 | ... | 962 | ... |
| 13 | 8 | East End Mothers' Home | 218 | 284 | 1,162 | 913 | 324 | 51 | 1,288 | ... |
| 1,370 | 1,025 | | 14,573 | 228,104 | 114,180 | 55,771 | 35,611 | 639 | 92,021 | 24,868 |
| | | DISPENSARIES. | | | | | | | | |
| ... | ... | Eastern | ... | 6,517 | 771 | 310 | 333 | 64 | 707 | ... |
| ... | ... | Hackney Provident | ... | 448 | 257 | 25 | ... | 221 | 246 | ... |
| ... | ... | London | ... | 2,091 | 433 | 124 | 269 | ... | 393 | ... |
| ... | ... | Queen Adelaide's | ... | 7,462 | 497 | 356 | 194 | 55 | 605 | 190 |
| ... | ... | Tower Hamlets | ... | 4,034 | 564 | 341 | 27 | 147 | 515 | ... |
| ... | ... | Whitechapel Provident | ... | 4,944 | 633 | 25 | ... | 556 | 581 | ... |
| 1,370 | 1,025 | | 14,573 | 253,600 | 147,335 | 56,952 | 36,434 | 1,682 | 95,068 | 25,058 |

### A SUMMARY OF THE WORK DONE IN 1894.

**It will be seen from the following summary that the Voluntary Hospitals and Medical Charities of London, during the twelve months ending 31st December, 1894, relieved over one million three hundred and eighty-three thousand patients at a cost of £699,527. The Ordinary Income only amounted to £559,725, leaving a deficiency of £139,802 on the year's work. The Legacies received in 1894 amounted to £137,134, being £13,668 less than in 1893, and £139,668 less than in 1892.**

| No. of Beds. | No. of Beds Daily Occupied. | HOSPITALS AND DISPENSARIES. | In-patients. | Out-patients. | Total Expenditure. | Income. | | | Total Income. | Legacies not included in previous column. |
|---|---|---|---|---|---|---|---|---|---|---|
| | | | | | | Charitable. | Proprietary. | Patients' Payments. | | |
| | | | | | £ | £ | £ | £ | £ | £ |
| 1,621 | 1,112 | Newington and South District ... | 15,701 | 194,526 | 104,924 | 44,584 | 33,691 | 13 191 | 91,466 | 9,069 |
| 566 | 417 | City and East Central District ... | 6,379 | 144,200 | 54,700 | 28.738 | 8,892 | 4,899 | 42.529 | 12,848 |
| 1,004 | 805 | Westminster District ... ... | 10,933 | 167,607 | 91,886 | 45,645 | 12,538 | 12,430 | 70.613 | 16,983 |
| 1,367 | 1,098 | St. Marylebone and West Central District ... ... ... ... | 11,498 | 197,367 | 110,856 | 52,278 | 20,829 | 9,576 | 82 683 | 30,765 |
| 1,662 | 1,436 | Kensington and West District ... | 15,833 | 199,484 | 144,131 | 70,071 | 30,529 | 9 377 | 109,977 | 35,211 |
| 801 | 597 | Islington and North-West District | 8,267 | 143,866 | 75,635 | 49,399 | 7,315 | 10,675 | 67,389 | 7,200 |
| 1,370 | 1,025 | Stratford and East-End District... | 14,573 | 253,600 | 117,335 | 56,952 | 36,434 | 1,682 | 95,068 | 25,058 |
| 8,391 | 6,490 | | 82,984 | 1,300,650 | 699,527 | 347,667 | 150,228 | 61,830 | 559,725 | 137,134 |

### OUR ILLUSTRATION!

### "JUST BEFORE BED TIME."

THE group which illustrates this page is a very typical one, showing, as it does, what children look like in hospital.

"Queen Victoria Ward," at the London Hospital, E., named after Her Majesty, and opened by her at her first visit to the East-end, has a history of its own. The number of children annually nursed there exceeds that of those treated in many "children's hospitals," and the cases form a splendid record of good results. The two great rooms are filled with children requiring active treatment and skilled nursing, and there is a background of solid hard work on the part of doctors and nurses behind this pretty foreground group.

Each ward forms a distinct nursery. and the two together contain 53 cots.

In the fine weather the street accidents are in a majority, on cold dark days bad burns and scalds help to fill the wards. In addition to the accidents a large number of surgical cases come into "Queen," as it is familiarly called. The boy in the long wooden "box splint" evidently belongs to the latter class. Fractured thighs are put into the same kind of apparatus, but patients with broken legs are kept strictly in bed; they do not share the change to the fireside in which other children can be indulged with impunity. Still, the "fractures" are not cases which need much commisseration, for boys and girls who break their legs are often healthy and high spirited, and after two or three days they get reconciled to their strict confinement to bed, and accept the splints philosophically. They find many things they can do, even whilst lying on their backs, and so much amusement can be derived from neighbours similarly circumstanced. Life in the wards is found to be as full of variety as life in the streets, and considerably easier for these little East-enders.

### THE LARGE NURSERY.

The attractions of the great nursery are manifold. First the children in their white night-gowns and scarlet flannel jackets claim notice, and then the toys! In the middle of the ward stands a fine rocking-horse, the admiration of all beholders; there are dolls, too, fine creatures, which remain as "ward property" after the distribution of the Christmas tree treasures. These are durable, but the smaller toys which sick children can play with in their beds need constantly renewing. Healthy children break things, and little people confined to bed work are still more destructive. Active brains and restless fingers must find employment in their waking hours, and therefore a liberal supply of cheap toys is gratefully received in a children's ward. "Unpainted and light" are the two essentials in such things, for heavy toys and clumsy scrap-books are wearying and take too much room in small beds, tempting children, for whom "lying flat" is most desirable, to wriggle themselves into constrained postures.

As for operations, when these are needed, they are performed by some of the cleverest surgeons of the day, and Royalty itself can only share the skill which is placed freely at the service of the poor in hospitals. These children in "Queen" have the same advantages precisely which a millionaire secures (with a big cheque) for his own afflicted child.

### ON ADMISSION.

"These cannot be children from the neighbourhood!" is the constant remark of visitors passing from the streets of Whitechapel into the pleasant wards, and failing to see any likeness between the teeming juvenile population of the bye-ways and these well-kept patients. On admission, dirt, rags, tangled hair, and uncut nails are such general characteristics, that a child who comes in clean is considered remarkable. Not only is the fair skin disguised with dirt, but there are numerous small sores and neglected abrasions of the skin, perhaps, from wretched boots, into which foreign substances have been rubbed. All these are carefully noted by the nurse who undresses the new case, and it is the attention to these small matters which the sufferer chiefly appreciates. The child may be indifferent to the results of a serious operation which he cannot understand, but he realises attention to his comfort.

## Figures and Tables

**Figure 1 f1:**
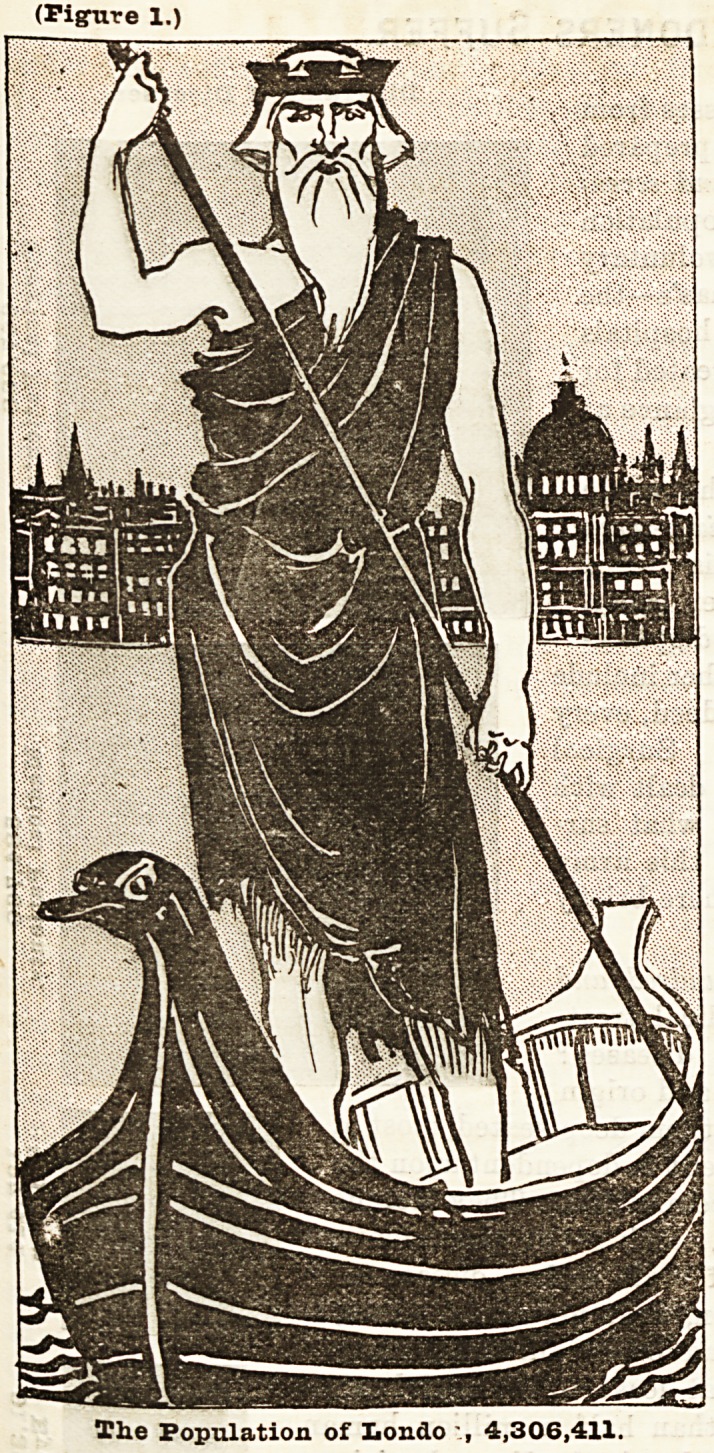


**Figure 2 f2:**
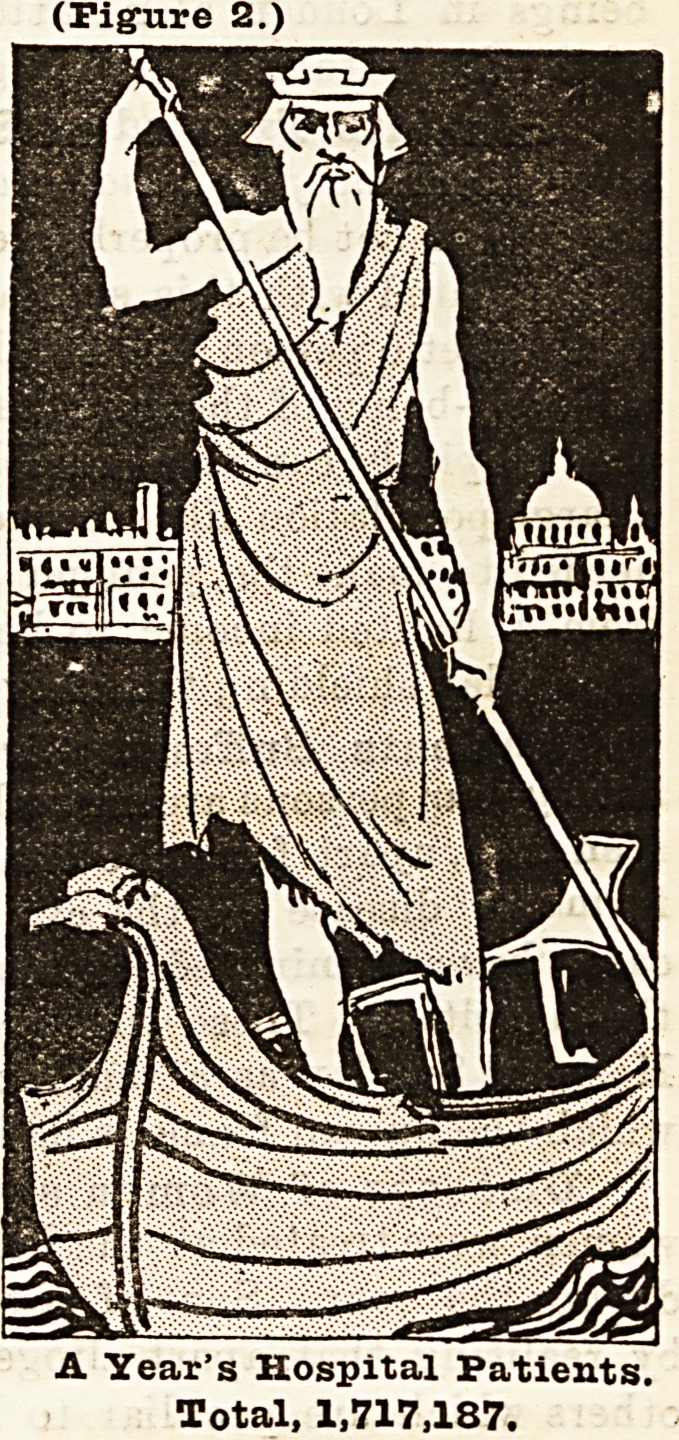


**Figure f3:**
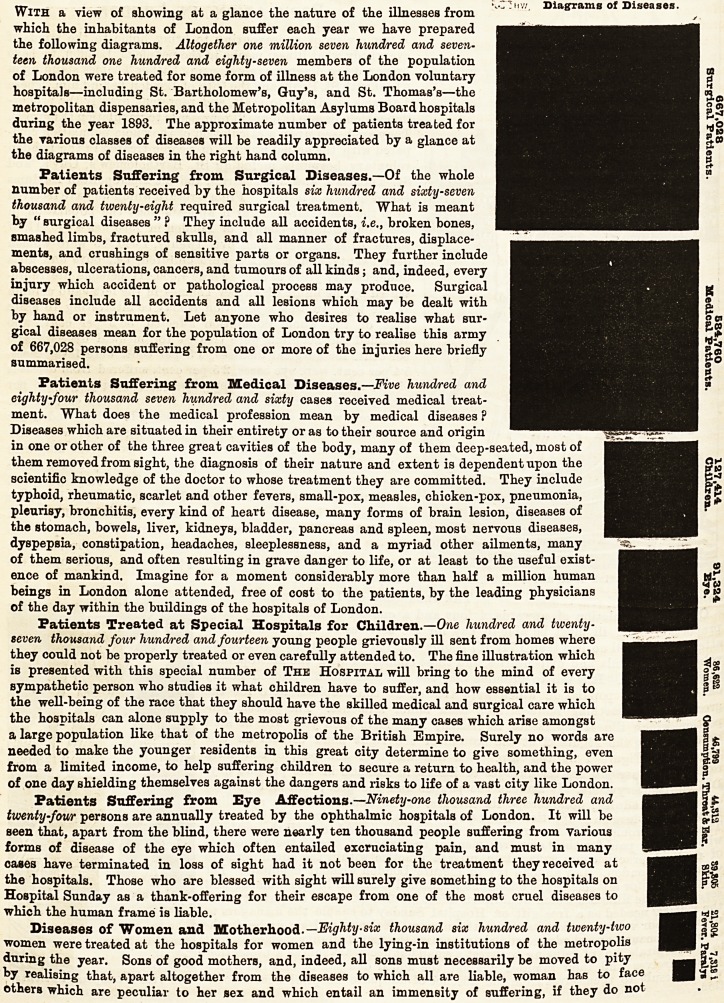


**Figure f4:**
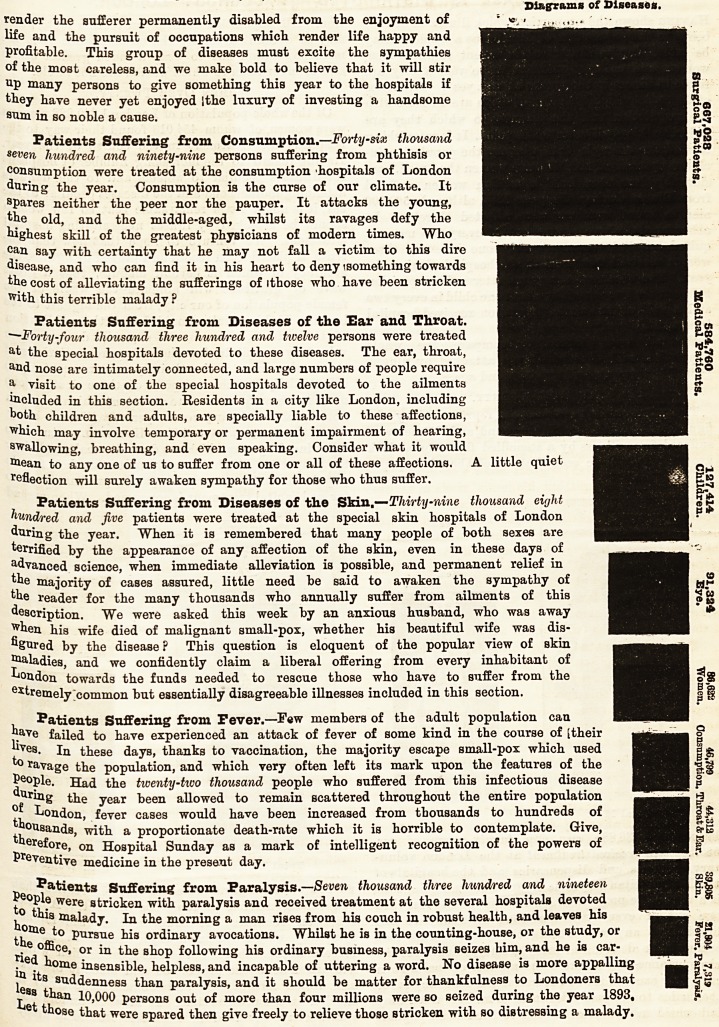


**Figure f5:**
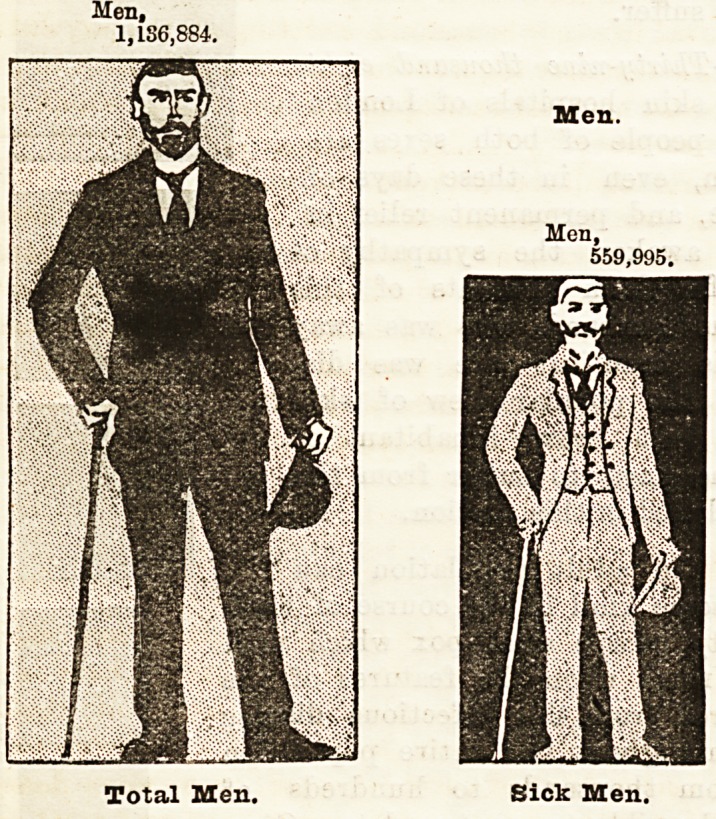


**Figure f6:**
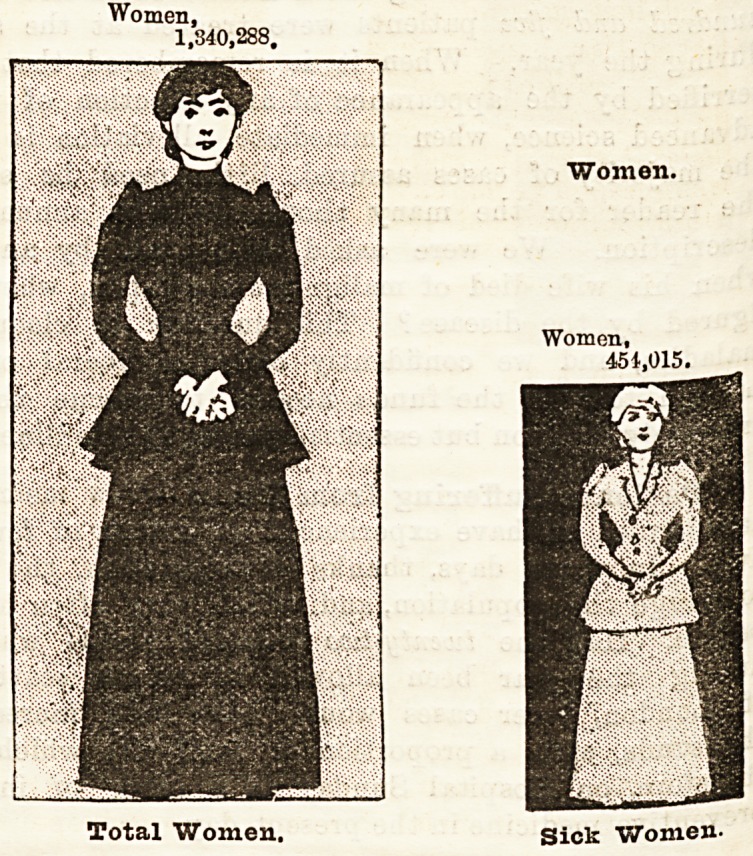


**Figure f7:**
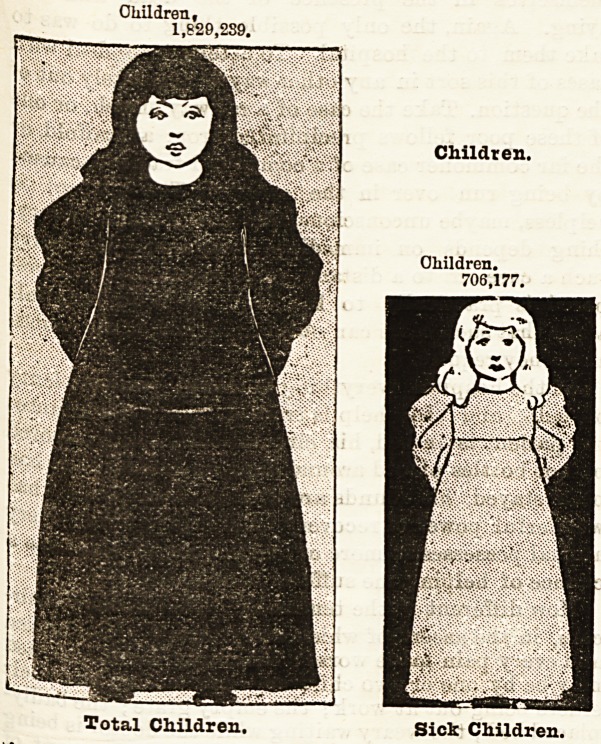


**Figure f8:**
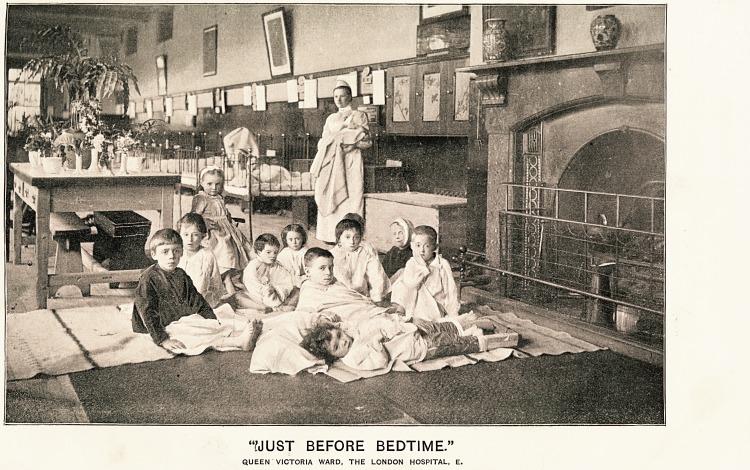


**Figure f9:**
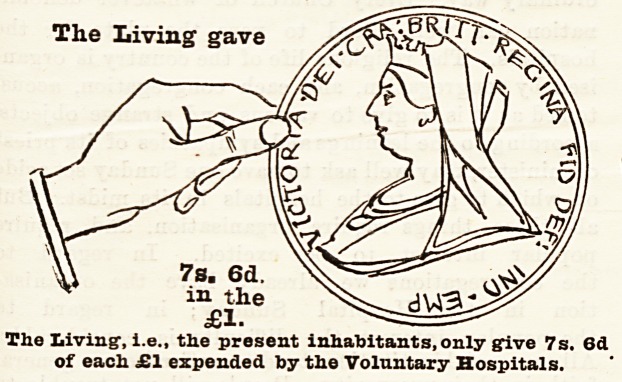


**Figure f10:**
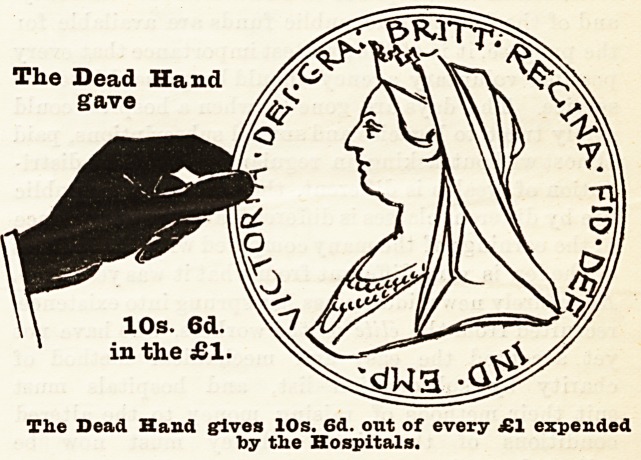


**Figure f11:**